# Supplementary material for: A Global Screen for Assembly State Changes of the Mitotic Proteome by SEC-SWATH-MS
Source: Cell Syst. 2020 Feb 26;10(2):133–155.e6. doi: 10.1016/j.cels.2020.01.001 (PMC7042714; doi:10.1016/j.cels.2020.01.001)
Supplement: Data S1. SEC-SWATH-MS Protein Chromatograms, Related to Figure 1 [file mmc6.zip › SECchrom_Q5EBL8_PDZ11_HUMAN_PDZD11_AIPP1_PDZK11_PISP_HSPC22.pdf]

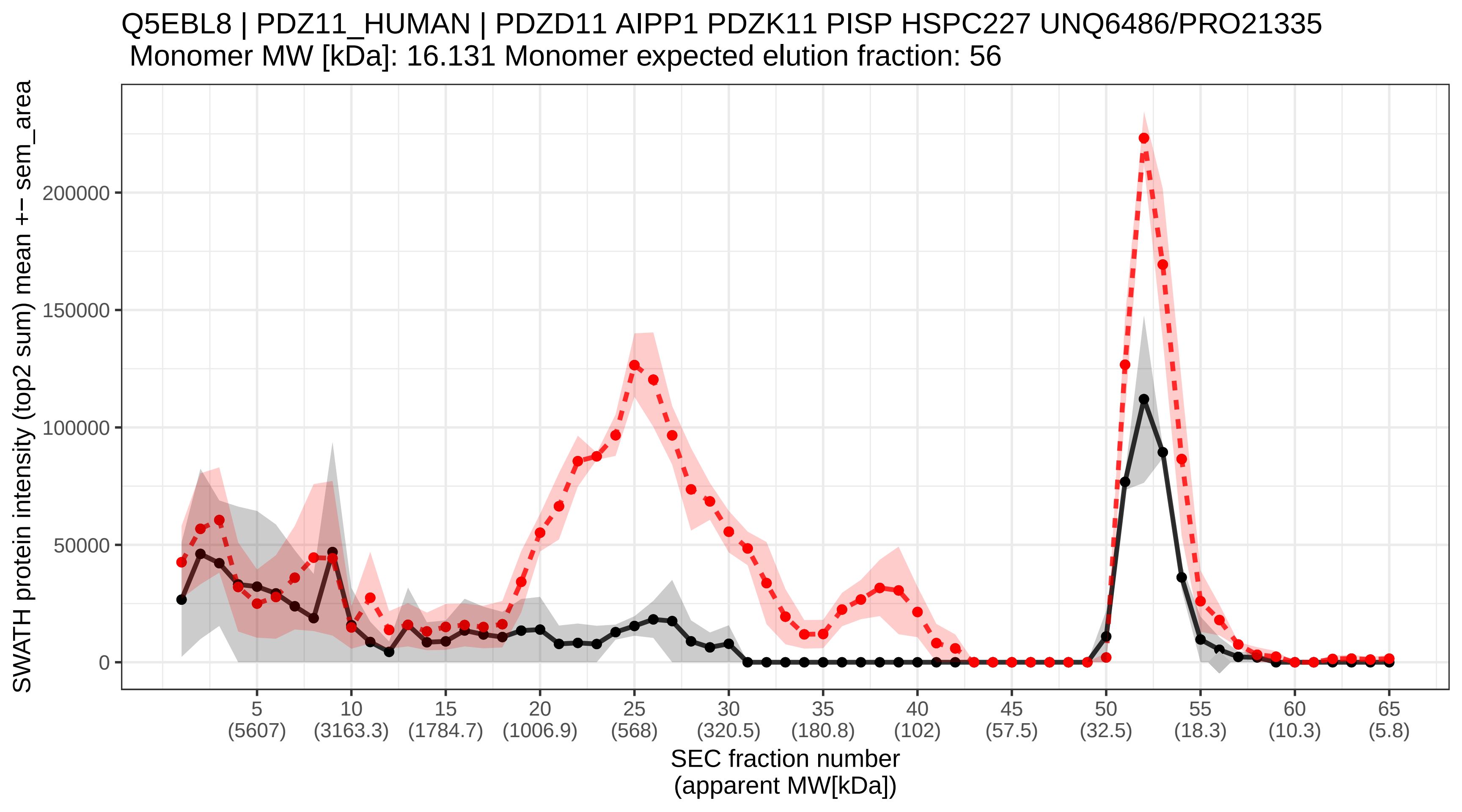

Q5EBL8 | PDZ11_HUMAN | PDZD11 AIPP1 PDZK11 PISP HSPC227 UNQ6486/PRO21335
Monomer MW [kDa]: 16.131 Monomer expected elution fraction: 56
SWATH protein intensity (top2 sum) mean +− sem_area
200000
150000
100000
50000
0
5
(5607)
10
(3163.3)
15
(1784.7)
20
(1006.9)
25
(568)
30
(320.5)
35
(180.8)
40
(102)
45
(57.5)
50
(32.5)
55
(18.3)
60
(10.3)
65
(5.8)
SEC fraction number
(apparent MW[kDa])
